# Subjective assessment of effectiveness, quality of life, and psychological status of patients receiving botulinum toxin therapy for hemifacial spasm, blepharospasm, or cervical dystonia

**DOI:** 10.20407/fmj.2019-027

**Published:** 2020-07-14

**Authors:** Tetsuharu Kako, Kazuya Nokura, Hiroshi Kaneko, Hideo Izawa

**Affiliations:** 1 Department of Neurology, Fujita Health University Bantane Hospital, Nagoya, Aichi, Japan; 2 Division of Psychosomatic Internal Medicine, Hoshigaoka Maternity Hospital, Nagoya, Aichi, Japan; 3 Department of Cardiology, Fujita Health University Bantane Hospital, Nagoya, Aichi, Japan

**Keywords:** Hemifacial spasm, Blepharospasm, Cervical dystonia, Botulinum toxin therapy, Quality of life

## Abstract

**Objectives:**

Evaluations of subjective effectiveness, quality of life (QOL), and mental status of patients receiving treatment with botulinum toxin (BTX) for hemifacial spasm (FS), blepharospasm (BS), and cervical dystonia (CD) were conducted using a self-administered questionnaire.

**Methods:**

Eighty-eight patients who received BTX treatment in the stable stage were analyzed. A numerical rating scale was used to assess treatment effectiveness, home QOL, and social QOL. Anxiety and depression were examined using the hospital anxiety and depression scale.

**Results:**

In men, the treatment effectiveness was 2.1±1.0 for FS patients, 2.8±0.5 for BS patients, and 4.0±2.0 for CD patients, which indicates that FS was more effectively treated than CD. QOL scores were higher and anxiety and depression scores were lower in FS patients than BS and CD patients. Overall, social QOL scores were lower than home QOL. A high prevalence ratio of depression was found in BS and CD patients.

**Conclusions:**

CD responded less effectively to BTX compared with FS and BS. Additionally, FS and BS patients exhibited similar treatment effects. All of these disorders affect the patient’s appearance, which can reduce self-esteem and social QOL and potentially cause anxiety and depression. BS and CD patients exhibited a higher prevalence of depression than FS patients, which indicates a relationship with the underlying mechanisms of dystonia. Asking patients about subjective effectiveness, QOL, and psychiatric status can help staff respond to patient issues.

## Introduction

Botulinum toxin (BTX) therapy is indicated for hemifacial spasm (FS), blepharospasm (BS), and cervical dystonia (CD). Among these disorders, BS and CD fall into the dystonia category, whereas FS has different underlying mechanisms. In the neurology department of our hospital, BTX is the main treatment for these three conditions. Although there are surgical options, many patients with these conditions have limited indications and do not wish to undergo surgery, and BTX therapy is their first choice.^1,2^ However, because the effectiveness of BTX treatment diminishes over 3–4 months, repeated treatment is necessary. FS involves acquired hypersensitivity as a result of disturbance of the facial nerve, resulting in involuntary spasm. Spasm is usually caused by compression, such as compression by the anterior inferior cerebellar artery on the facial nerve, or by sequelae, such as Bell’s palsy. BTX therapy is a recommended treatment for FS.^3^ Conversely, BS is exhibited as involuntary closure of the eye due to focal dystonia, caused by abnormalities in basal ganglia circuitry. Involuntary movements often extend to other parts of the face.^1,2^ CD is included in head and neck dystonia, as is BS. Head deviations, such as rotation and lateral bending, are observed intermittently and regularly. Symptoms are likely to be induced by certain movements. Light touching by a hand on the face or head is a sensory trick that can improve symptoms.^4^

These three conditions not only reduce patients’ quality of life (QOL) because of impaired movement, but also because these conditions impact the patient’s appearance, potentially causing anxiety and depression. Dystonia, on the other hand, is sometimes mistakenly considered to be psychogenic because the true cause is unknown,^5^ and because of comorbidity with psychiatric problems.

In order, FS, BS, and CD patients are increasingly refractory to treatment in daily practice. We conducted a self-reported survey among FS, BS, and CD patients to confirm our observations in a clinical setting. We investigated differences between each disease group after patients underwent a subjective evaluation of therapeutic effects, QOL, and mental state.

## Methods

A self-administered questionnaire survey was conducted with patients who received BTX treatment at the Fujita Health University Bantane Hospital during the 4 months from September 7 to December 28, 2018. Patients who received three or more injections and exhibited stable symptoms were selected. The effectiveness score, QOL at home (home QOL), and QOL in social settings (social QOL) were evaluated using a numerical rating scale. QOL was divided into two categories because stress related to the patient’s appearance or physical problems may differ between being at home and being in public. Treatment effectiveness also was measured using a questionnaire-based method, defined as described below: the degree of severity was 10 for the most severe symptoms and 0 for no symptoms. Thus, the higher the treatment effectiveness, the lower the score. For QOL, the highest QOL score was 10 and the lowest was 0. The Japanese version of the HAD scale^6–8^ was used to evaluate anxiety and depression. According to the original version, the scores of seven items about anxiety and seven items about depression were summed. Scores of zero to seven points were classified as no anxiety or depression, scores of 8 to 10 points were classified as possible anxiety or depression, and scores of 11 or more points were classified as a definite diagnosis of anxiety or depression. Furthermore, the latter two were combined and analyzed. The actual scale used is shown in the figure. The original HAD scale is not shown because of copyright restrictions.

The research protocol was evaluated and approved by the ethics committees of the Fujita Health University School of Medicine (registration no. HM19-340). Written informed consent was obtained from patients regarding publication of this report and accompanying data.

### Statistical analyses

Data were analyzed using EZR.^9^ Data were compared among the three disease groups, focusing mainly on the five categories, age, and gender. For clarity, all data are presented as means±standard deviation, even for the non-normal distribution data. To compare the age and HAD scale scores for three groups, a one-way analysis of variance (ANOVA) test was performed, and Tukey’s multiple comparisons test (TK) was added for significant differences. The chi-square test and Fisher’s test with pairwise comparisons (PW) were used for categorical variables. To compare numerical values such as effectiveness score and QOL score in three groups, the Kruskal–Wallis (KW) test was performed, and the Steel–Dwass multiple comparison (SD) test was added for those with significant differences. To compare numerical values in paired groups the Wilcoxon signed-rank test (WS) was performed. A p-value of less than 0.05 was considered to indicate a significant difference.

## Results

Of the 91 patients who met the criteria, three were not enrolled because of the effects of dementia. The remaining 88 (96.7%) patients were included in the analysis. Of the 88 patients, data for 36 patients with FS (21 female and 15 male, chi-square test, p=NS), 27 patients with BS, and 25 patients with CD were analyzed. BS was significantly more common in women than in men (23 women and four men; chi-square test, p=9.6×10^–7^). CD was significantly more common in men than in women (seven women and 18 men; chi-square test, p=0.0019) (Table 1). The mean age (years) was 61.7±13.1 for FS patients, 64.6±11.4 for BS patients, and 51.7±12.6 for CD patients, and the mean age of CD patients was significantly lower than that of FS and BS patients (one-way ANOVA; p=0.000877, TK p=0.0081, p=0.0010). When the data were divided by gender, the same pattern was also shown in women, but there was no significant difference in men.

We first analyzed the gender-combined data (Table 2). The treatment effectiveness scores were 2.9±2.0 for FS patients, 3.3±2.1 for BS patients, and 4.0±2.1 for CD patients. There were no significant differences in each disease group. Home QOL scores were 7.6±2.0 for FS patients, 6.6±1.9 for BS patients, and 5.9±2.4 for CD patients. CD patients exhibited significantly lower home QOL scores than FS patients (KW; p=0.011, SD; p=0.016). Anxiety scores were 5.3±3.7 for FS patients, 7.2±4.8 for BS patients, and 7.9±4.7 for CD patients. There were no significant differences in each group. Depression scores were 4.8±3.4 for FS patients, 6.5±3.5 for BS patients, and 7.8±4.4 for CD patients, and CD patients exhibited significantly higher values than FS patients (ANOVA; p=0.012, TK; p=0.0098). In FS patients, home QOL values were significantly higher than those of social QOL (WS: p=0.0021).

We then performed analyses after separating the patients by gender (Tables 3 and 4). In men, the treatment effectiveness scores were 2.1±1.0 for FS patients, 2.8±0.5 for BS patients, and 4.0±2.0 for CD patients, and FS patients exhibited significantly lower values compared with CD patients (KW; p=0.0085, SD; p=0.0093). Home QOL scores were 7.9±2.0 for FS patients, 6.8±1.9 for BS patients, and 6.1±2.3 for CD patients. CD patients exhibited significantly lower values for home QOL compared with FS patients (KW; p=0.037, SD; p=0.035). In women, anxiety scores were 5.0±3.9 for FS patients, 7.7±5.0 for BS patients, and 9.7±3.3 for CD patients. In CD patients, anxiety scores were significantly higher than those of FS patients (ANOVA; p=0.031, TK; p=0.046). Depression scores were 5.0±3.6 for FS patients, 6.9±3.5 for BS patients, and 8.6±3.5 for CD patients. In CD patients, depression scores were significantly higher than those of FS patients (ANOVA; p=0.031, TK; p=0.046).

Total scores for home QOL and social QOL for the three disease groups are shown in Table 5, for each gender. In patients of both genders, home QOL scores were significantly higher than those for social QOL (WS; men: p=0.026, women: p=0.031).

According to the original HAD scale, we analyzed the prevalence rate of anxiety or depression disorder by dividing the criteria into no, possible, and definite. We also analyzed the data by combining the latter two (possible and definite). Although there were no differences in the prevalence of anxiety among the three disease groups, CD and BS patients exhibited a significantly higher prevalence of depression than FS patients (Fisher’s exact test, p=0.0073, PW; p=0.015, p=0.048) (Table 6).

## Discussion

In the current study, there were significantly more women than men with BS and significantly more men than women with CD. In accord with these findings, Wakakura et al. reported a high rate of cases of BS among women.^10^ Additionally, another previous study reported that, among patients with dystonia, gender differences change with age; many male CD patients were between 30 and 40 years old, and many female BS patients were between 50 and 60 years old.^11^ Moreover, CD was more common in young men, whereas BS was more common in older women.^11^ The current findings were similar to the results of these previous studies. A study of many cases in other Asian regions indicated that BS is significantly more common among women compared with men.^12^

Clinical trials of BTX in Japan have reported improvement rates of 74.5% for FS, 89.9% for BS, and 41.6% for CD.^13–15^ Because the subjects in the current study were treated with BTX regularly, our results may have differed from those of previous studies. CD is thought to be intractable compared with BS and FS. Our data revealed gender differences, indicating that CD was less likely to improve in men compared with than women. However, we believe that this finding may have been related to the lower level of muscle strength in women. FS and BS have been reported to exhibit similarities in various background factors despite the different underlying mechanisms.^16,17^ The current study also revealed similar results between patients with FS and BS.

Regarding patient QOL, we assumed that there would be a difference between home QOL and social QOL. The results indicated that social QOL values were lower than those of home QOL in all disease conditions, but a significant difference was only found in the FS group. When we analyzed the three disease groups together, home QOL scores were significantly higher than social QOL scores. These findings suggest that patients may have difficulty working or participating socially due to loss of self-esteem or physical problems.

Patients with BS reported significantly lower visual-related QOL than those with facial spasm, and anxiety and depression were also significantly severe.^18^ According to a previous study by Wakakura et al., 33.6% of patients with BS exhibited severe QOL decline, which was proportional to severity. Additionally, mood disorders were observed in 60.2% of patients with BS, but there was no correlation with severity.^10^

BS can cause mood disorders and decreased QOL even in patients with mild symptoms.^10^ In the current study, the measured values for both anxiety and depression increased in the following order: FS, BS, and CD. Depression scores were significantly higher in CD patients than in FS patients. In women, CD patients scored significantly higher than FS and BS patients in both anxiety and depression. However, in contrast to our prediction, the results revealed no differences between FS and BS patients. A detailed examination of psychological examinations for FS and BS reported no significant differences, and a mental burden was observed even in FS patients.^17^ Additionally, a previous report suggested that only obsessive-compulsive disorder was significantly higher among patients with BS.^19^ Hall et al. reported that anxiety-related factors were twice as high for BS patients compared with FS patients.^16^ It has also been reported that non-motor symptoms such as anxiety and depression improve with BTX treatment-related improvement of symptoms.^16,20,21^ In the current study, the FS group included only a few cases with post-facial nerve paralysis and our results suggest that the severity may have been milder than samples of FS patients reported in previous studies. Several previous studies suggest that dystonia has a high lifetime incidence rate of anxiety, depression, and obsessive-compulsive disorder.^19,22–24^ The current study is consistent with these previous findings, revealing a high prevalence rate of depression among BS and CD patients. Thus, it is important not to overlook the presence of depression and to consider potential causal relationships between depression and dystonia.

Unfortunately, we did not examine obsessive-compulsive disorder in the current study. However, our results for anxiety and depression are similar to those reported previously. One previous study suggested that BS was not closely related to anxiety.^25^ Basal ganglia abnormalities have been suggested as risk factors for both BS and obsessive-compulsive disorder.^24^

Clinicians should be aware that patients with BS and CD have not only motor but also psychiatric symptoms. Elucidating whether dystonia is caused by psychological factors or whether dystonia causes mental illness is challenging. Thus, clinicians should be careful to avoid concluding that motor symptoms in patients with dystonia are psychogenic based on psychiatric symptoms seen in the course of the patient. Improving understanding of obsessive-compulsive disorder in daily clinical practice is an important challenge for future studies.

Asking patients about their subjective improvement can be a substitute for performing time-consuming and difficult movement evaluations. Confirming patients’ subjective QOL assessment can make it easier to respond to patient issues. Patients undergoing BTX treatment are likely to exhibit impaired self-esteem and reduced QOL and may require psychological assistance.

The most important limitation of the current study is that we did not include evaluations before and after intervention with BTX treatment. In the future, a more comprehensive prospective survey with a larger sample size should be conducted.

## Figures and Tables

**Figure 1:**
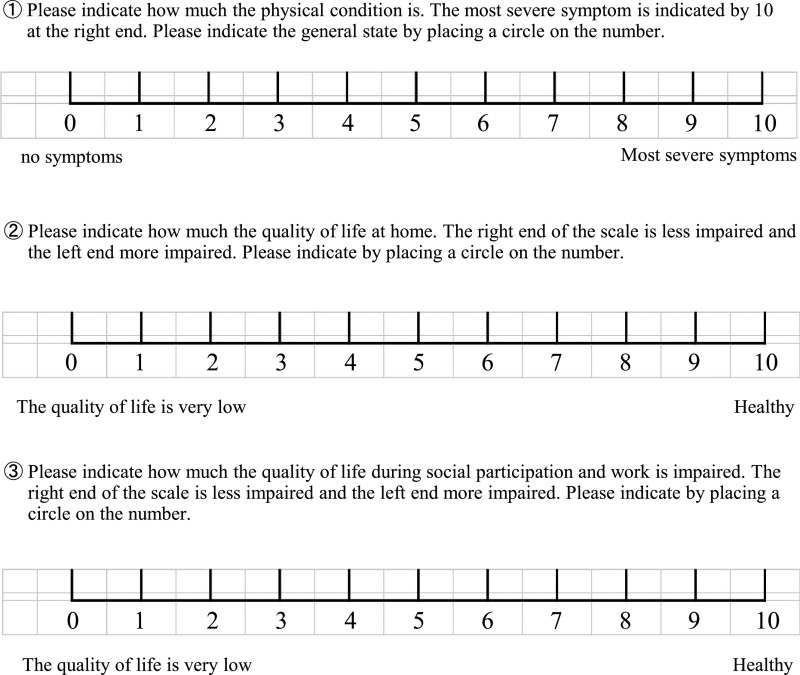
Questionnaire document (in Japanese) that was presented to the patient to complete.

**Table 1:**
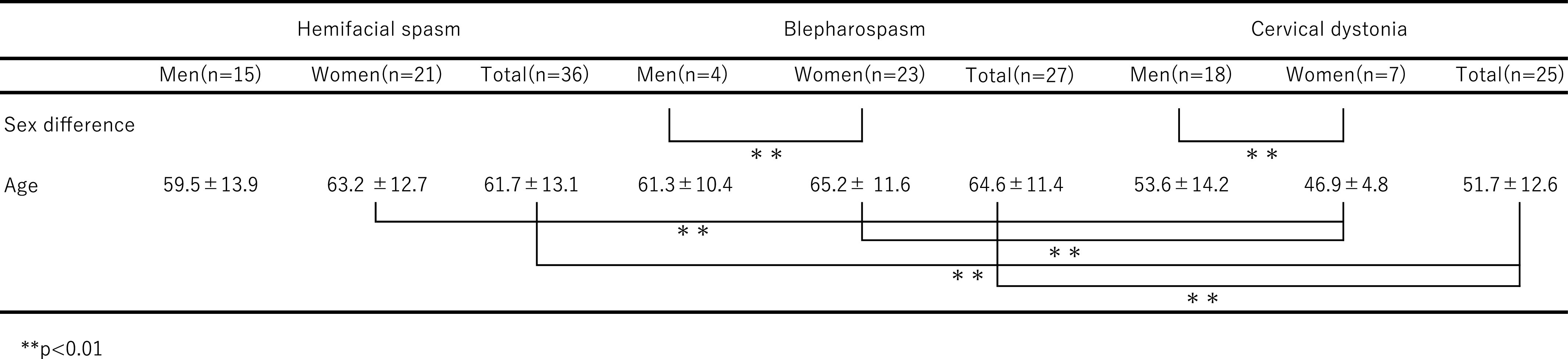
Average age in each disease group and each gender group

**Table 2:**
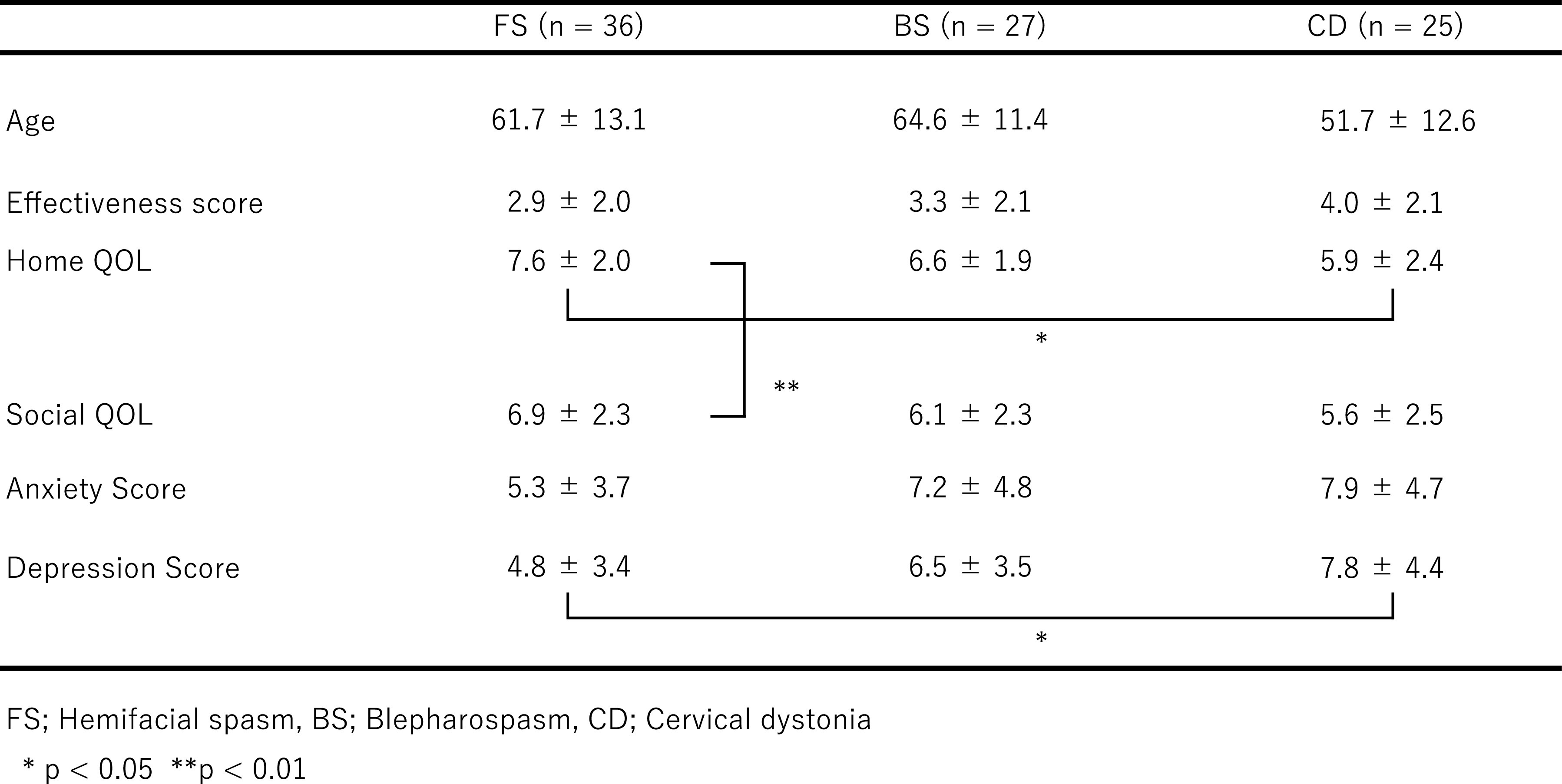
Gender-combined data

**Table 3:**
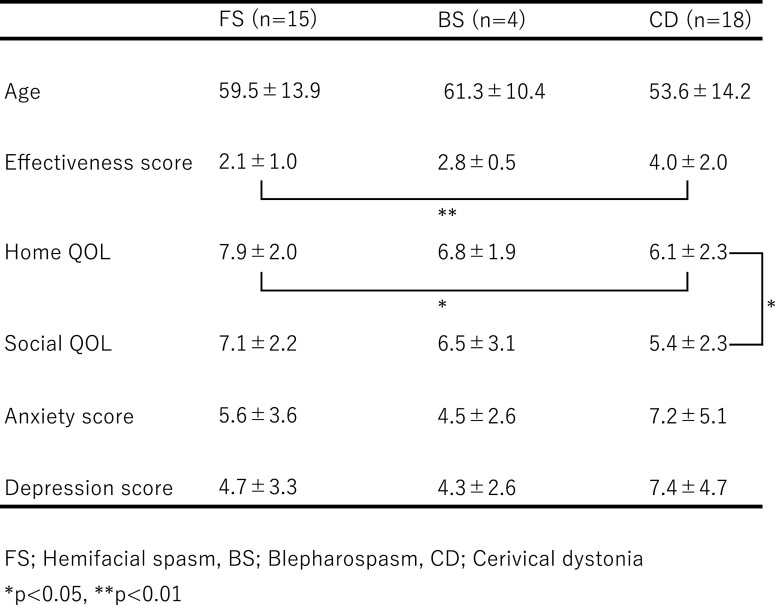
Results from male patients

**Table 4:**
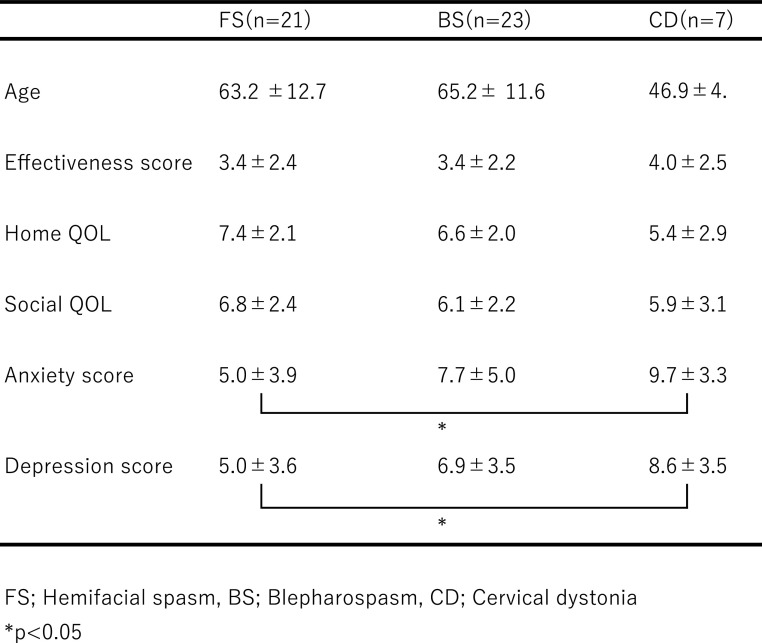
Results from female patients

**Table 5:**
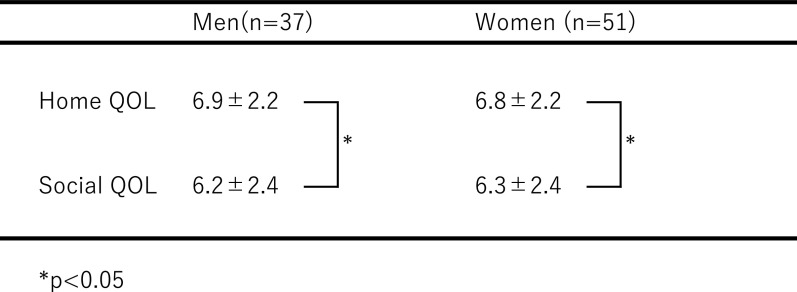
Home QOL and social QOL scores in each gender group

**Table 6:**
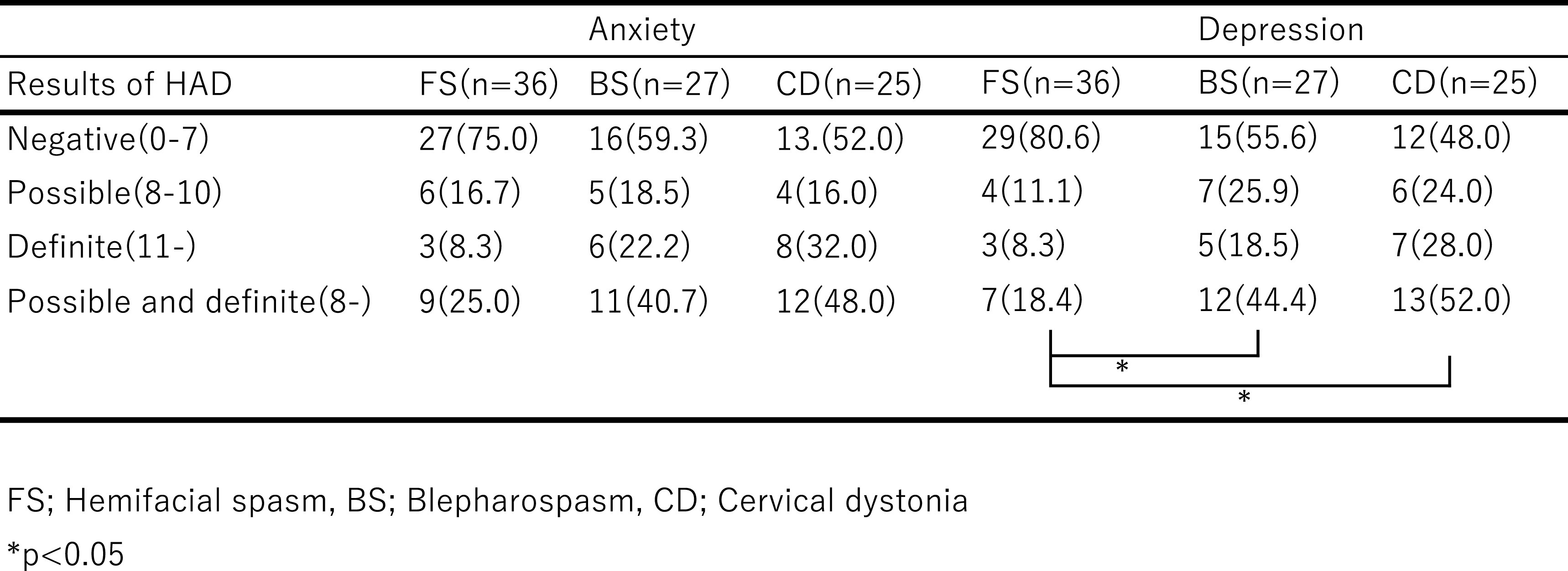
Patient sample composition according to HAD results (%)
